# Esteemed Colleagues: A Model of the Effect of Open Data on Selective Reporting of Scientific Results

**DOI:** 10.3389/fpsyg.2021.761168

**Published:** 2021-10-21

**Authors:** Eli Spiegelman

**Affiliations:** Economics and Social Sciences, CEREN, EA 7477, Burgundy School of Business – Université Bourgogne Franche-Comté, Dijon, France

**Keywords:** open data, signaling game model, research ethics, esteem, replication crisis, replication crisis in psychology, academic dishonesty behaviors, academic dishonesty and misconduct

## Abstract

Open data, the practice of making available to the research community the underlying data and analysis codes used to generate scientific results, facilitates verification of published results, and should thereby reduce the expected benefit (and hence the incidence) of p-hacking and other forms of academic dishonesty. This paper presents a simple signaling model of how this might work in the presence of two kinds of cost. First, reducing the cost of “checking the math” increases verification and reduces falsification. Cases where the author can choose a high or low verification-cost regime (that is, open or closed data) result in unraveling; not all authors choose the low-cost route, but the best do. The second kind of cost is the cost to authors of preparing open data. Introducing these costs results in that high- and low-quality results being published in both open and closed data regimes, but even when the costs are independent of research quality open data is favored by high-quality results in equilibrium. A final contribution of the model is a measure of “science welfare” that calculates the ex-post distortion of equilibrium beliefs about the quality of published results, and shows that open data will always improve the aggregate state of knowledge.

## Introduction

Experimental work in the social sciences is currently undergoing a replication crisis (Ioannidis, 2005; Stevens, 2017; Obels et al., 2020). The Open Science Collaboration (2015) successfully replicated 36 out of 100 experiments published in high-ranking psychology journals; Camerer et al. (2016, 2018) find reproducibility rates of around 61% in economics experiments. In a survey of 1,500 scientists, Baker (2016) found that 70% had failed to replicate another researcher’s results, and 50% had failed to replicate their own. There are many potential sources of these phenomena, but one of the most direct is that researchers are being less than completely forthright about the nature of the results they publish. Their incentives to do so are clear: on the “demand side,” tenure and promotions, successful grant proposals, and even informal esteem from colleagues are all examples of how researchers get some utility from the perception of having done important work, whether or not such perceptions are rigorously supported by the data. Furthermore, on the “supply side,” the inherent complexity of interpreting empirical data implies that the “true” result is rarely completely unambiguous. Even setting aside cases (which nevertheless do exist) of outright fraud or fabrication of data, it may often be possible for otherwise principled and honest researchers to lean on their results as it were, engaging in gentle falsification, or “p-hacking,” for instance through selective analysis or reporting of results.

Even “partial dishonesty” can have negative social effects, as it generates an unwarranted image of the state of scientific knowledge. For instance, gender differences in risk aversion, with females less willing to take risks than males, long represented a “stylized fact” that emerged from studies designed to address other questions. Publishing confirmatory results lent credibility to such papers by showing that they fit with the existing body of knowledge, but also perpetuated a particular description of the social nature of gender. However, a meta-analysis by Filippin and Crosetto (2016) subsequently showed that the effect was, if not illusory, then much more fragile than had previously been estimated. Subsequent verification of previous work in this sense represents scientific progress and at the same time a progressive view of gender.

Perhaps the central assumption of this paper is that such “fact-checking,” systematically applied to the accumulated body of published results, should act as a kind of disciplining tool on what gets published in the first place: researchers may be tempted to inflate the “importance” of their results in order to acquire a certain esteem from the research or wider community, but a downward revision of the importance induces an esteem penalty, so it is preferable to honestly present results of minor importance, rather than being caught in such inflation or falsification. A potential lever to encourage the disciplining verification is *open data*, which refers to the practice of making the underlying data and analysis codes used to generate results available to the research community, along with the paper itself. This clearly facilitates verification; so long as it also increases the probability of some third party actually engaging in such verification, it should thereby reduce the expected benefit (and hence the incidence) of p-hacking and other forms of academic dishonesty. The very top journals in many fields, for instance in economics, psychology and marketing, require open publication of data and analysis codes with the paper. However, the requirement is far from systematic. For instance, at the time of this writing 9 of the top 20 economics journals merely “encouraged” open data submissions. Furthermore, such encouragement is not generally effective (Tenopir et al., 2011). Alsheikh-Ali et al. (2011) investigated 500 published papers coming from high impact journals from various scientific fields, finding that only 9% had their raw data stored online publicly. Womack (2015) reached a similar conclusion; from a sample of 4,370 papers published in 2014 in the highest impact journals, only 13% made their data publicly available online.

The idea that researchers are motivated to publish “important” results due to a mechanism of esteem indicates a link to signaling models, which form the basis of the theoretical construction in this paper. The signal structure has several inter-related layers, which are developed sequentially. First, the presentation of the published paper itself should be considered as a signal of the underlying quality of the scientific result obtained. This is modeled as a relatively “cheap” signal: “authors” in the model are privately informed of the quality of their results, and can present them as whatever they choose. “Readers” are motivated to identify dishonest presentation, although verification is costly. Section 2 shows that in equilibrium, as might be expected, the lower this cost, the more verification—and the less falsification—occurs. The second layer of signaling is the choice of open or closed data, that is, of high or low verification costs. Intuitively, a “nothing to hide” principle choosing high verification costs should be taken as a bad signal, and indeed Section 3 shows that a case where the author can choose a high or low verification cost regime (that is, open or closed data) results in unraveling. All high-quality results, which require no falsification, will be published in open data, which allows readers to identify any result published in closed data as being of low quality, making falsification impossible.

These results are promising, but seem to conflict with the empirical patterns described above in which adoption of open data is very low. In this regard, a potentially important second kind of cost not included in the model is the cost to authors of securely and accessibly storing their data in open repositories. Surveys have shown that this process is perceived as a significant barrier to researchers in opening their data (Stodden, 2010; Marwick and Birch, 2018; Chawinga and Zinn, 2019). Section 4 of the paper extends the model to incorporate these costs as well, assuming that they distribute idiosyncratically across authors, and independently of the quality of results obtained. The main result is that high- and low-quality results will be published in both open and closed data regimes, but that open data will be favored by high-quality results. The structure of the equilibrium implies that the falsification among the low-quality results published in the open-data regime is *higher* than it would be in a single, high-cost (closed) regime. However, a final contribution of the model is a measure of “science welfare” that calculates the ex-post distortion of equilibrium beliefs about the quality of published results, and shows that open data will always improve the aggregate state of knowledge. The paper finishes with a discussion of these results in the context of the literature on open data in the social sciences.

## Selective Reporting and Verification Given Verification Costs

### Interaction Structure: The Prestige Game

The interaction is called a *prestige game*, indicating the interpretation of the utility functions that benefit is largely determined by the equilibrium beliefs about the quality of a piece of research produced. The game has two players: an author *A* and a representative reader *B*. The author does some research, reaching a result of stochastic quality *q*. For simplicity, suppose that there are two possible qualities *H* and *L*, represented as real numbers with *H* > *L*, and probability *p* of reaching result *H*. The quality is not directly observable, but is represented through the published paper; we denote by $\hat{q}$ the published description of the quality, and write it with lower case to distinguish it from the true quality of the result. That is, $\hat{q}\in \left\{h,l\right\}$, where *h* and *l* are taken to conventionally indicate *H* and *L*, respectively. In the standard manner of games of incomplete information, it will sometimes be convenient to refer to *A* players who observe *q* = *H* as being of *type A*_*H*_, and those who observe *q* = *L* as *A*_*L*_. *A*’s utility is therefore the prestige, or esteem that she experiences, or more concretely the expected value of *q*, upon announcing a quality of $\hat{q}$.

Denote *s*_*q*_ ≔ $\,\mathrm{Pr}\left[\hat{q}=h|q\right]$, the probability that a result of *q* is represented as if it were *H*. This means that *s*_*L*_ is probability of the kind of selective analysis or reporting alluded to above, that inflates results, or makes a low-quality result appear to be higher than it is. The model abstracts from the cost of engaging in this falsification (and in practice, inflation probably takes no more effort on *A*’s part than would an “honest” analysis); the focus is on *B*’s choice to look more deeply into the results. Specifically, at cost *k*, the reader *B* may verify *A*’s work; denote the probability that *B* does so as *v*.^1^

Assume this verification process correctly identifies *q* to the public. If it turns out that $\hat{q}$≠*q*, then *B* gets some esteem (benefit) and if $\hat{q}>q$, then *A* suffers a cost. The purpose of these assumptions is to reflect the social processes of prestige following the revision of scientific results. The basic assumptions are

• A downward revision ($\hat{q}>q$) generates a “large” cost *C* to *A*, and for simplicity awards the same benefit to *B*.
• An upwards revision ($\hat{q}<q$) has no inherent effect of *A*, other than the revision of the perceived quality itself, and awards a “small” benefit ε to *B*.
• If there is no revision of quality, there is no effect on *A*’s esteem, although *B* must still pay the cost *k*.

To this point, the model has five parameters, which will be supposed to be in the order *H* > *L* > *C* > ε > *k*. The order of *L* and *C* is not important, but both benefits of verification must be greater than the cost *k* or non-verification is trivial. Although the model is simpler if *C* ≥ *L*, which means that it is at least as good not to write a paper at all as to have a low-quality result be revealed as deceptive, the results below are based on the less restrictive, inverse case. If *B* does not verify the results of the research, the quality is taken to be its equilibrium average, conditional on $\hat{q}$.

**FIGURE 1:**
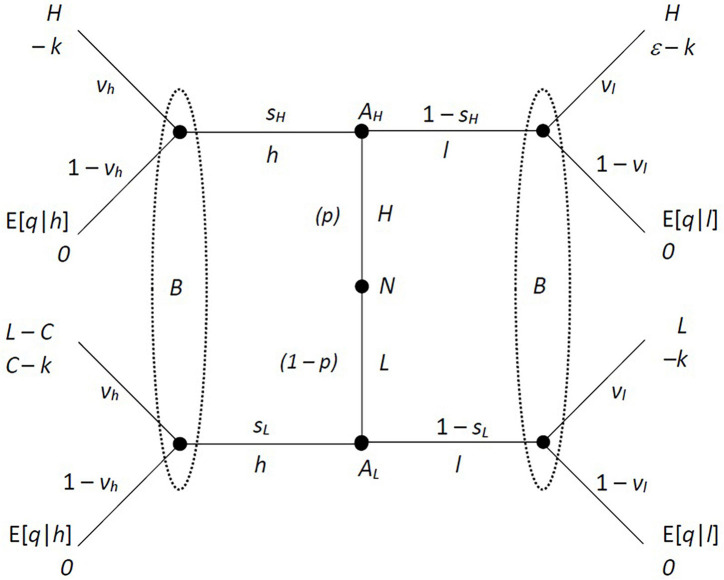
Extensive form of the basic game structure.

This basic game can be represented as a special case of a signaling game, as in the game tree in Figure 1. Here Nature moves first at the central node, choosing the quality of the research *H* or *L*. Then *A* moves, choosing a quality to declare, *h* or *l*. Finally, *B* decides whether to verify the results (with probability *v*), or not [with probability (1 – *v*)]. The solution concept will be a sequential equilibrium of this game, allowing for mixed strategies. The appearance of expected values in the payoffs is the only departure from standard theory.

### Equilibria

Signaling games are generally characterized by three sets: a set *T* of types representing the private information of a message sender, a set *M* from which signals may be drawn, and a set *V* of possible actions the message receiver may use in response. An equilibrium consists of strategies from *T* to *M* and from *M* to *V*, together with a set of beliefs over *T* given the realization of the message, such that each strategy is a best response to the other taking the beliefs into account, and the beliefs are consistent with the signaling strategies, following Bayes’ Rule where possible. In this model, clearly *T* = {*H*, *L*}, *M* = {*h*, *l*}, *V* = {verify, do not}, and the beliefs are induced by


$$\mathrm{Pr}\left[q=H|\hat{q}=h\right]=\frac{p\,s_{H}}{p\,s_{H}+\left(1-p\right)\,s_{L}}$$

$$\mathrm{Pr}\left[q=H|\hat{q}=l\right]=\frac{p\,\left(1-s_{H}\right)}{p\,\left(1-s_{H}\right)+\left(1-p\right)\,\left(1-s_{L}\right)},$$

so long as these are defined.

It may be noted that this model does not satisfy the so-called *single-crossing property*, a simplification common in signaling games which generates a sorting of sender types, so “higher” types always send weakly “higher” messages in equilibrium. While it will always be the case that for *A*_*H*_, being verified is at least as good as not being verified, while for *A*_*L*_ getting verified is (weakly) always worse, *B* prefers to verify *A*_*H*_ after a message of *l* and *A*_*L*_ after a message of *h*.^2^ That is, while the single-crossing property holds with respect to *B*’s actions, it does not hold with respect to the messages that induce those actions.

An important distinction among signaling models differentiates cheap talk, in which utility does not depend directly on the message sent, from costly signaling in which message senders can demonstrate something concerning their type directly through the signal sent. In the context modeled here, the message itself has no costs; however, the effect of *B*’s choice on *A* does depend directly on the message sent. The general form of the utility function $U_{A}\,\left(q,\hat{q},v\right)$ therefore is not generally constant in $\hat{q}$ (and in particular not when *v* = 1), so this can be considered a model of “impure” cheap talk. The single-crossing property is maintained in canonical models of cheap talk through a fixed and common-knowledge “bias” of the sender’s preference with respect to the receiver’s, meaning a divergence between the sender’s (type-dependent) preferred action and the optimal action for the receiver to take, conditional on sender type. While the preferred action depends on the type of the sender, that is, the degree of “conflict” is constant; a key result is that the lower the degree of conflict, the more information may be transmitted in equilibrium. Another way of seeing the violation of the single-crossing property in the current model is that the bias depends on *A*’s type. *A*_*H*_ has preferences not particularly at odds with those of *B*, while *A*_*L*_ has clear incentives to dissemble.

Finally, the fact that *A*’s utility when *v* = 0 depends directly on beliefs is important mainly at the level of interpretations. In standard models, these beliefs are instrumental, and matter because they generate behavior that impacts utility. However, these reactions are an (optimal) mapping from the beliefs generated by the different strategies in equilibrium. In the current case, this mapping is direct: the utility to a researcher of having published a particular result is defined as the perception of its quality. This means that message choice by one type of *A* imposes a kind of externality on the other if verification does not occur, but the effect can be thought of as a continuous “choice” by *B* of how to interpret each signal. Of course, there is no inherent incentive involved in this “choice of beliefs,” other than the restriction imposed by Bayes’ rule, but formally speaking, whether the choice is determined by optimal behavior or by application of Bayes’ rule is irrelevant to *A*.

Equilibrium requires a mapping from *q* to the signal from *A*; from the signal to verification from *B*; and a set of beliefs over *A* types following each possible signal. The beliefs are defined as above. Each *A*-type optimal strategy consists of a simple decision rule, while *B* has a rule for each possible signal received. It is easy to see the following:

*A*_*H*_ chooses *s*_*H*_ = 1 if


(1) $$v_{h}\,H+\left(1-v_{h}\right)\,E\,\left[q|h\right]\geq v_{l}\,H+\left(1-v_{l}\right)\,E\,\left[q|l\right],$$

while for *s*_*L*_ = 1, *A*_*L*_ requires that


(2) $$v_{h}\,\left(L-C\right)+\left(1-v_{h}\right)\,E\,\left[q|h\right]\geq v_{l}\,L+\left(1-v_{l}\right)\,E\,\left[q|l\right].$$

Concerning *B*, verification of a signal *h* requires


(3) $$\frac{\left(1-p\right)\,s_{L}}{p\,s_{H}+\left(1-p\right)\,s_{L}}\,C\geq k$$

while for the signal *l* the threshold is


(4) $$\frac{p\,\left(1-s_{H}\right)}{p\,\left(1-s_{H}\right)+\left(1-p\right)\,\left(1-s_{L}\right)}\,\varepsilon \geq k.$$

Supplementary Appendix 1 investigates the equilibria of this game. These are described in Result 1, below

*Result 1: equilibria of the prestige game*

*Consider the game described above, and suppose in addition that* $p<\frac{C}{C+\varepsilon }$. *Then there are three equilibrium components, depending on k.*

A. *If k* ≥ (1 − *p*) *C*, *then there are no separating equilibria, but any mixed strategy profile s_*H*_* = *s_*L*_* = *s*ε *[0, 1] can stand as an equilibrium, with no verification of any results by B.*
B. *If ktextless* (1 − *p*) *C, then there is a unique semi-separating equilibrium in which s_*H*_* = *1; s_*L*_* = $\frac{p}{1-p}\,\frac{k}{C-k}$*; v_*h*_* = $\frac{\left(H-L\right)\,\left(1-\frac{k}{C}\right)}{\left(H-L\right)\,\left(1-\frac{k}{C}\right)+C}$*; and v_*l*_* = *0. This equilibrium will be known as the* k-game.
C. *If k* < *p*ε, *then there is also a pooling equilibrium in which s_*L*_* = *s_*H*_* = *0, and v_*l*_* = *v_*h*_* = *1.*

**FIGURE 2:**
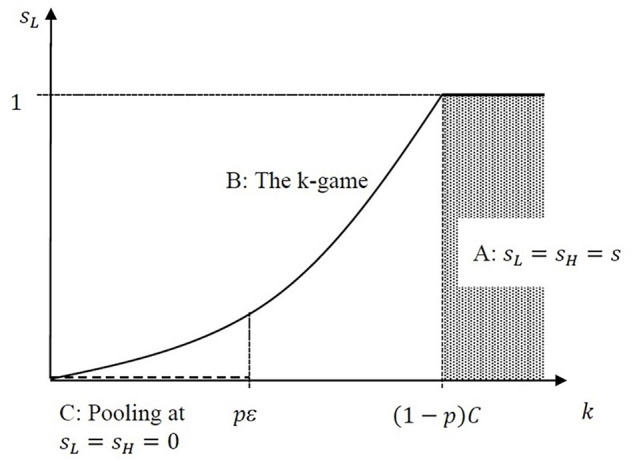
Equilibria of the prestige game. Dashed line indicates pooling equilibria for very low values of *k*, and shaded area indicates that any pooling behavior can stand as an equilibrium if *k* is high enough.

Figure 2 illustrates the equilibrium falsification rate as *k* changes.

The additional restriction on *p* in Result 1 rules out a somewhat perverse set of equilibria in which the probability of a high-quality result is large enough that, in equilibrium, is it those declared as $\hat{q}=l$ that appear “suspicious,” and are preferentially investigated. This does not seem to correspond to the real-world situation of scientific publishing, and to the extent that the *C* is “large” and ε is “small,” the ratio in the condition will be close to unity, so the restriction is relatively mild.

More interesting are the effects of changes in *k* on the kinds of equilibria that exist. First, part A describes a situation in which the cost of verification is “too high.” In particular, *B* does not verify any “babbling” equilibrium, where both types of *A* choose the same strategy, so the signal is uninformative. As a result, any such non-communicative strategy profile stands as an equilibrium. The esteem awarded to any signal is *pH* + (1 – *p*) *L*, so neither type of *A* has any reason to deviate, although if *s* = 1, then an Intuitive Criterion argument along the lines of Cho and Kreps (1987) could lead *A*_*H*_ to deviate, choosing *s*_*H*_ = 0 if *B* expected this.^3^

Part B of Result 1 describes the *k-game*, which will be the central focus of the analysis below. If *k* < (1−*p*)*C*, then *B* will verify a babbling equilibrium concentrated on the signal *h*, driving *A*_*L*_ – but not *A*_*H*_ – away from that strategy and generating separation. The separation can’t be complete, though, or *B* would stop verifying, leading *A*_*L*_ to move back in. Therefore, the form of the equilibrium is in semi-separation, with *A*_*H*_ always sending the message *h*, while *A*_*L*_ mixes, sometimes sending the deceptive signal *h* and otherwise the honest one, *l*. Since in this equilibrium all results announced as *l* are actually of quality *L*, *B* verifies only the signal *h*. The average esteem to *A*_*L*_ is precisely *L*, with the costs of exposure (when verified) after sending message *h* exactly balancing out the benefits of deception (when not verified). While the closed-form solution is not intuitive, it is easy to see that the esteem to *A*_*H*_, by contrast, which amounts to *v*_*h*_*H* + (1 − *v*_*h*_) *E*[*q*|*h*], is strictly between *H* and *L*; as standard in games of incomplete information, deceptive behavior by the “low” types exerts an externality on the “high.”

Finally, part C states if *k* < *p*ε, then *B* will verify a degenerate signal profile on either message.^4^ Unlike the all-*h* profile, by contrast, the all-*l* one does stand as an equilibrium. *A*_*H*_ gets the payoff of *H*, and *A*_*L*_ gets the payoff of *L*. The former can do no better, and so long as the off-path beliefs also threaten verification should the latter deviate to signal *h*, *A*_*L*_ strictly prefers the equilibrium action. Notice that these off-path beliefs are not defined in equilibrium, but for instance, any sequence of “trembles” such that both types of *A* have equal probability of deviating in each element of the sequence will generate a sequential equilibrium of the same form. This equilibrium will not be the focus in what follows. It has a somewhat interesting interpretation as a “possible world” of scientific publishing; authors are “modest,” never claiming high importance of their results, and readers systematically check all results, arriving at a complete state of knowledge about each. In this sense it seems like a “healthy” state of affairs, although it does impose costs on *B*. However, it is not particularly realistic as a description of the field, and moreover the equilibrium is not very robust to changes in the model. In particular, it relies on *A*_*H*_ being indifferent between claiming a high importance and having it revealed by *B*’s verification. To the extent that there might also be some esteem from recognizing the importance of one’s own work, or “embarrassment” from presenting results of *q* = *H* with the label *l*, *A*_*H*_ should also suffer a cost when verified, which lead *A*_*H*_ to deviate from this equilibrium.

### Comparative Statics: Result of Changes in *k*

The interpretation of open data in this model is to reduce the cost of verifying results; data sharing reduces *k*, and the model therefore gives some predictions about how open data might affect behavior. We see immediately that at least in the k-game, as *k* falls, *v*_*h*_ rises and *s*_*L*_ falls. Keeping in mind the possibility of a “corner solution” (part A of Result 1), this can be stated as follows

*Corollary 1: so long as it reduces k to less than C (1 – p), open data will reduce the degree of inflation of weak results due to selective reporting.*

*Corollary 2: if open data reduces the level of inflation of weak results, it also increases the degree of verification.*

An interesting implication of these corollaries concerns what might be termed *science welfare*. The goal of science is to have as accurate a picture of the functioning of the world as possible. To this extent, inflation of results, which distorts the impression that readers have of their significance, can be seen as reducing the overall quality of the scientific endeavor. While a scalar measure of “quality” does not directly map to distortion of the message contained in the results, it seems plausible that in presenting a “low quality” result of *q* = *L* with the signal *h*, researchers will also change its overall message or real-world implications. Of course, it is common knowledge in the model that the unconditional probability of a high-quality result is *p*; rational expectations in equilibrium ensure that overall this remains the case. Moreover, the signal *l* perfectly identifies low-quality results in the *k*-game, so from a scientific point of view there is no problem there. On the other hand, the public “state of knowledge” following a signal of *h* is not equal to *H*, which can be interpreted as a science welfare loss.

Following this interpretation, define a *science welfare loss function* as the *rate of unverified inflation of results*. A simple form for this is


(5) $$W=-\left(1-v_{h}\right)\,s_{L}.$$

Expression (5) indicates that either full verification or fully honest reporting would be enough to reduce the science welfare loss to zero. Since *v*_*h*_ falls and *s*_*L*_ rises with *k*, the final corollary of this section can be stated

*Corollary 3: reducing the costs of verification increases science welfare.*

## The Effect of Different Possible Levels of *k*

The results above show that imposing open data, if it sufficiently reduces the cost of verifying existing results, should unambiguously improve the quality of research publication. What happens when *k* is a choice by *A*? This is the scenario where the journal or discipline does not require open data, but allows authors to opt into it. This is modeled as allowing *A* to select from a set of possible values of *k*, called *regimes*. To model shared or unshared data, suppose that there are two regimes, *k*_*o*_ < *k*_*c*_, indicating that *o*pen data has a lower verification cost than does *c*losed data. Suppose both regimes are parametrized such that the *k*-game is the equilibrium played. The choice of regime is made after Nature decides on *q*, simultaneously with $\hat{q}$.^5^

The structure of the interaction in any regime is identical to the prestige game described in section “Introduction,” and as mentioned, the focus will largely be on *k*-game equilibria in each regime, or *k*-game components to the “double-game” equilibrium including regime choice. In this respect, the principal element that this new layer of regime choice adds to the strategic context is to endogenize the probability that a paper is of quality *H* in any regime; this value will now be determined by the equilibrium distribution of A-types that select into each regime. Define *φ*(*k*) as the probability that a paper under regime *k* is of quality *H*. That is, if authors finding quality *H* choose *k* with probability λ*_*k*_*(*H*) and papers of quality *L* choose *k* with probability λ*_*k*_*(*L*), then


(6) $$\varphi \,\left(k\right)=\frac{p\,\lambda_{k}\,\left(H\right)}{p\,\lambda_{k}\,\left(H\right)+\left(1-p\right)\,\lambda_{k}\,\left(L\right)}.$$

This value of *φ*(*k*) will replace *p* in each regime *k*, with the rest of the prestige game analysis following as in section “Introduction.” Supposing that the parameters are such that, in each regime, the *k*-game would be played in isolation, the following holds

*Result 2: Equilibrium under regime choice*

*Consider a 2-regime prestige game where the k-game conditions hold in each regime, k_*o*_ < k_*c*_, and define λ and φ as above. In any equilibrium*

A.
λko⁢(H)=1 *;* $\frac{p}{\left(1-p\right)}\,\frac{k_{o}}{C-k_{o}}<\lambda_{k_{o}}\,\left(L\right)\leq 1$

B. *Behavior follows the k-game in the k_*o*_ regime; A_*L*_ always plays l in k_*c*_*
C. *Off-path beliefs mimic the k-game in the k_*c*_ regime*

Result 2 establishes an “unraveling” effect under free regime choice. *A*_*H*_ strictly prefers the open to the closed regime, both because verification is more frequent there, and because the expected value of unverified messages is higher. Only the second of these is an advantage for *A*_*L*_, of course, and so low-quality types follow high into the open regime only up to the point of indifference by *B*. As a result, the open regime contains a mix of types, and the *k-*game is played there, while only *L*-quality results are ever published in the closed regime. This then further implies that falsification is impossible in the closed regime, and so all those who enter it (honestly) declare *l*. However, the result that only the *l*-signal is made in the closed data equilibrium means that the equilibrium does not determine beliefs following a signal of *h* in the closed regime. Interestingly, several plausible off-path beliefs—for instance that only *A*_*H*_ (or only *A*_*L*_) would choose this signal—turn out to upset the equilibrium. However, the equilibrium can be maintained with beliefs that re-create the *k*-game that would have been played in that regime, if *A*_*H*_ did not deviate toward the open data. These off-path beliefs also admit an interesting interpretation in terms of “non-stigmatization.” In essence, while the equilibrium never has high-quality results in the *k*_*c*_ regime, it relies on the belief that this “could happen” off the equilibrium path, and so signals of type *h* in *k*_*c*_ are not systematically verified. On the other hand, *B* would also ascribe such a “tremble” to *A*_*L*_ types with some probability, so some verification occurs. In other words, the unobserved, counterfactual declarations of *h* in the high-cost regime are not given particularly bad (or good) interpretations in the model.

Another interesting feature of this model concerns the indeterminacy noted in *A*_*L*_’s strategy in part A. Overall, the rate of falsification in the *k*_*o*_ regime must be such that *B* is indifferent between verifying or not, but any combination of entry to that regime and falsification once there that generates this overall rate can stand. Consider generally the falsification rate in each regime (subscripted by regime rather than by *q* since only *q* = *L* results in falsification in the *k*-game equilibrium):


$$s_{k}^{*}=\frac{\varphi \,\left(k\right)}{1-\varphi \,\left(k\right)}\,\frac{k}{C-k}$$

This can be combined with (6) to give


(7) $$s_{k}^{*}=\frac{p}{\left(1-p\right)}\,\frac{k}{C-k}\,\frac{\lambda_{k}\,\left(H\right)}{\lambda_{k}\,\left(L\right)}.$$

Expression (7) implies that the more *H*-quality papers select into a regime relative to *L* in equilibrium, the more the *L*-quality papers in that regime will falsify their results. It is tempting to interpret this as “trying to fit in with a better pool”; the equilibrium falsification rate must leave *B* indifferent between verifying or not. *L*-quality papers have to falsify more in equilibrium as the relative frequency of *H* increases, in order to balance out increased risk to *B* of paying the cost *k* without getting any benefit. Because in the specific equilibrium of Result 2, λko⁢(H)=1, moreover, this means that *A*_*L*_’s equilibrium strategy can be determined up to


(8) sk*o⁢λko⁢(L)=p1-p⁢koC-ko,

and any combination of the terms on the left that satisfy (8) are equivalent for the equilibrium. The entry and falsification rates are jointly determined, in other words, but the overall level of falsification—and therefore verification—in the open-data regime is the same, whether it represents a large fraction of the *A*_*L*_ types falsifying to a moderate extent, or a smaller fraction falsifying more consistently.

This is important because it implies that the expected value of an unverified signal *h* does not change with *λ_*k*_*. Recall from the formula in Result 1 (B) that the verification rate does not depend on *p*, which intuitively is because this rate serves in equilibrium to leave *B* indifferent between signals conditional on having observed *q* = *L*. The same holds in the two-regime setting. So long as neither λ_*k*_ (*L*) nor λ_*k*_ (*H*) are equal to zero,


E⁢[q|h,k]=L⋅Pr⁢[L|h,k]+H⋅Pr⁢[H|h,k]

$$=L\,\frac{s_{k}\,\left(1-p\right)\,\lambda_{k}\,\left(L\right)}{s_{k}\,\left(1-p\right)\,\lambda_{k}\,\left(L\right)+p\,\lambda_{k}\,\left(H\right)}+\text{H}\,\frac{p\,\lambda_{k}\,\left(H\right)}{s_{k}\,\left(1-p\right)\,\lambda_{k}\,\left(L\right)+p\,\lambda_{k}\,\left(H\right)}$$

$$=L\,\frac{\frac{p}{\left(1-p\right)}\,\frac{k}{C-k}\,\frac{\lambda_{k}\,\left(H\right)}{\lambda_{k}\,\left(L\right)}\,\left(1-p\right)\,\lambda_{k}\,\left(L\right)}{\frac{p}{\left(1-p\right)}\,\frac{k}{C-k}\,\frac{\lambda_{k}\,\left(H\right)}{\lambda_{k}\,\left(L\right)}\,\left(1-p\right)\,\lambda_{k}\,\left(L\right)+p\,\lambda_{k}\,\left(H\right)}$$

$$+\text{H}\,\frac{p\,\lambda_{k}\,\left(H\right)}{\frac{p}{\left(1-p\right)}\,\frac{k}{C-k}\,\frac{\lambda_{k}\,\left(H\right)}{\lambda_{k}\,\left(L\right)}\,\left(1-p\right)\,\lambda_{k}\,\left(L\right)+p\,\lambda_{k}\,\left(H\right)}$$

$$=H-\frac{k}{C}\,\left(H-L\right).$$

Stated plainly, adding a low-*k* regime to a costlier one may result in different *A* types choosing different regimes, and if it does, then the equilibrium effects of this will be balanced by changes in the falsification rates in each regime. But the verification rate in any regime (provided it maintains its *k-*game structure in equilibrium) will not change with the addition of another regime.^6^

*Remark 1:*

*The equilibrium results of selection of A-types into different regimes include adjustment of falsification rates, with higher rates in the regime containing more A_*H*_ types; it does not affect the verification rates in either regime, compared to the single-regime k-game.*

Combined with the unraveling result, this implies that, while there is a continuum of equilibria, with some fraction of *A*_*L*_ between zero and $\frac{p}{\left(1-p\right)}\,\frac{k_{o}}{C-k_{o}}$ choosing the high-cost regime (closed data), the low cost regime absorbs the falsification, and the science welfare is not affected by which equilibrium occurs. This follows directly from Remark 1. Science welfare was defined as the overall rate of unverified falsification, and neither of those quantities (verification rates or overall falsification) are affected in this model by the addition of a high-cost regime that attracts only *A*_*L*_.

*Result 3: Science welfare in the two-regime, free-choice model is determined by the costs of the lower-cost regime.*

## Costs of Preparing Open Data

The model from section “The Effect of Different Possible Levels of *k*” has some interesting characteristics, but is ultimately not quite satisfactory. It induces a correlation between regime choice and quality, suggesting that results published in open data should be, on average, of higher quality than others. But it at once predicts a multiplicity of equilibria with respect to *A*_*L*_’s regime choice, and also quite starkly that in any of them, all results published in the high-cost regime should be declared as low-quality, and the “unraveling” in terms of science welfare is complete. In addition, while the “no-stigmatization” result is anecdotally interesting, the off-path beliefs are at once arbitrary, imposed for no other reason than supporting the equilibrium, and rather precise, requiring a specific relationship between two different kinds of deviation. An extension that ensures that *A*_*H*_ may sometimes opt for the *k*_*c*_ regime even in the presence of multiple *k*-games “solves” many of these issues, yielding sharper predictions with more intuitive interpretation, at the cost of an additional assumption and parameter.

In surveys, one of the principal reasons that researchers cite for not participating in open data is the time and effort costs of doing so (Stodden, 2010; Marwick and Birch, 2018; Chawinga and Zinn, 2019). In the model so far, on the other hand, the choice of regime has been costless. Suppose, therefore, that there are still two possible levels of *k*, *k*_*o*_ < *k*_*c*_, and that each determines a separate *k*-game into which authors select. In addition, there is a utility penalty *K* to player *A* for choosing *k*_*o*_ due to the time and effort costs of opening the data. The goal of this assumption is to make it so that some, but not all, of the *A*_*H*_ players choose the *k*_*c*_ regime, so it requires idiosyncratic costs to generate the differences. For simplicity more (perhaps) than realism, suppose that *K* distributes across *A* players randomly according to a continuous distribution *G*(*K*) that is independent of *q*. For notational convenience, also normalize *H – L* = 1.

These assumptions induce a change in the equilibrium structure. Intuitively, *A* players of both types with high enough costs choose the closed regime, while those with low costs choose the open. This is driven by higher verification rates in the open regime, which make it preferable to *A*_*H*_, and therefore increase the prestige (expected value) of unverified publications there. However, if there is a cost to entering the open regime, and the benefit is conditional on either being an *A*_*H*_ type or not being verified, then there is no reason why *A*_*L*_ would ever choose that regime and then announce *l*. In short, for *A*_*L*_, the strategy (*k*_*c*_, *l*) dominates the strategy (*k*_*o*_, *l*), and rather than announcing *l* in the *k-*game of the open regime, *A*_*L*_ goes to the closed one. This implies that all publications in the open regime are announced as *h*. On the other hand, while this change appears to affect behavior in important ways, the informational content of the equilibrium can be preserved, as *k*-game structure of the open data regime is maintained by the rate of entry to the regime, rather than the rate of falsified signaling within it. Dominance of the closed-data regime for results announced as *l* eliminates one of *A*_*L*_’s strategic margins to allow probabilistic verification by *B*, and hence the multiplicity of equilibria found above, but the other strategic margin remains available, preserving the basic game intuition. This is summarized in Result 4.

*Result 4: Consider a two-regime environment with k_*o*_ < k_*c*_ and idiosyncratic costs of entry to the k_*o*_ regime. Then*

A. *There is a unique set of equilibrium entry rates to the open regime, which satisfies* λko⁢(H)>λko⁢(L)

B. sko=1 *for both A_*H*_ and A_*L*_*
C. *The k-game is played in the closed regime among the residual, high-K A-types*

Part (B) of Result 4 follows from the dominance argument above. Part (C) follows from the presence of both types of *A*-player in the closed regime. To see part (A), note that if *B* does not verify, then *A*_*L*_ will enter if costs are low enough, while if *B* always verifies, then *A*_*L*_ will never enter. Therefore *B* must be indifferent to justify probabilistic verification. Building from expression (7), this implies that it must be that in equilibrium


(9) $$1=\frac{p}{\left(1-p\right)}\,\frac{k_{o}}{C-k_{o}}\,\frac{\lambda_{k_{o}}\,\left(H\right)}{\lambda_{k_{o}}\,\left(L\right)}.$$

Expression (9) shows that in equilibrium, more high-type authors choose the open regime than closed, justifying the inequality in part (A). Furthermore, it shows that entry in equilibrium must be in a fixed ratio. The unique level at which this ratio can stand in equilibrium is determined by threshold values $\left(K_{H}^{*},K_{L}^{*}\right)$ such that (9) holds when (λko⁢(H),λko⁢(H))=(G⁢(KH*),G⁢(KL*)), and also


(10) $$v_{o}H+\left(1-v_{o}\right)E\left[q|h,k_{o}\right]=EU\left[k_{c}|H\right]+K_{H}^{*}$$

(11) $$v_{o}\left(L-C\right)+\left(1-v_{o}\right)E\left[q|h,k_{o}\right]=EU\left[k_{c}|L\right]+K_{L}^{*}$$

Expressions (10) and (11) indicate that for each type *T* = *H*, *L* of *A*, there is a threshold cost $K_{T}^{*}$ such that those with cost greater than $K_{T}^{*}$ choose the closed regime, while those with lower costs choose the open. The extra cost of data preparation must be exactly balanced by a higher expected payoff in the open regime for both types at this threshold.

It is clear from inspection of (10) and (11) that any level of *v*_*o*_ will determine a pair $\left(K_{H}^{*},K_{L}^{*}\right)$. Moreover, since, as *v*_*o*_ rises from zero to one, the left-hand side of (10) rises, while that of (11) falls, the difference or ratio between the implied levels of $K_{H}^{*}$ and $K_{L}^{*}$ is monotonic in *v*_*o*_. Thus, there can be only one level of *v*_*o*_ that also satisfies the specific ratio determined in (9). To see that there is at least one, notice that first that (9″) implies that


(9″) $$G\,\left(K_{L}^{*}\right)=\frac{p}{\left(1-p\right)}\,\frac{k}{C-k}\,G\,\left(K_{H}^{*}\right)<G\,\left(K_{H}^{*}\right)⟶K_{L}^{*}<K_{H}^{*}$$

Next, Remark 1 implies that in the *k*-game in the closed regime, *EU* [*k*_*c*_ | *L*] = *L*, while


$$EU\left[k_{c}|H\right]=\frac{\left(H-L\right)\,\left(1-\frac{k_{c}}{C}\right)}{\left(H-L\right)\,\left(1-\frac{k_{c}}{C}\right)+C}H+\frac{C}{\left(H-L\right)\,\left(1-\frac{k_{c}}{C}\right)+C}\left[H-\frac{k_{c}}{C}\left(H-L\right)\right]$$

$$EU\left[k_{c}|H\right]=\frac{\left(1-\frac{k_{c}}{C}\right)}{\left(1-\frac{k_{c}}{C}\right)+C}H+\frac{C}{\left(1-\frac{k_{c}}{C}\right)+C}\left[H-\frac{k_{c}}{C}\right]$$

$$EU\left[k_{c}|H\right]=\frac{\left(1-\frac{k_{c}}{C}\right)}{\left(1-\frac{k_{c}}{C}\right)+C}H+\frac{C}{\left(1-\frac{k_{c}}{C}\right)+C}H-\frac{C}{\left(1-\frac{k_{c}}{C}\right)+C}\frac{k_{c}}{C}$$

$$EU\left[k_{c}|H\right]=H-\frac{k_{c}}{\left(1-\frac{k_{c}}{C}\right)+C}>L$$

Inserting these values into (10) and (11) and investigating the boundary conditions, we see that when *v*_*o*_ = 0,


(10″) $$E\left[q|h,k_{o}\right]=H-\frac{k_{c}}{\left(1-\frac{k_{c}}{C}\right)+C}+K_{H}^{*}$$

(11″) $$E\left[q|h,k_{o}\right]=L+K_{L}^{*}.$$

Combining these implies that


(12) $$K_{H}^{*}-K_{L}^{*}=L-\left[H-\frac{k_{c}}{\left(1-\frac{k_{c}}{C}\right)+C}\right]<0.$$

The inequality in expression (12) means that there are “too many” *A*_*L*_ types entering the open regime when verification is “low enough.” Specifically, the threshold cost for *A*_*L*_ is higher than that for *A*_*H*_, which means that the ratio would be greater than unity, and cannot be accommodated in (9). On the other hand, the implicit threshold of $K_{L}^{*}$ hits zero when verification is equal to its (single-regime) equilibrium level in the open regime, as then the expected value to *A*_*L*_ of both regimes equals *L*. This is clearly “too few” *A*_*L*_-types entering. Because (10) and (11) are both continuous in *v*_*o*_, there must therefore be a single level of entry that satisfies all conditions, establishing the result.

Regarding science welfare in this configuration, as is intuitive, the costs to using open data, or more exactly the resultant distortions they induce, increase equilibrium falsification relative to the model in section “The Effect of Different Possible Levels of *k*.” But it is interesting to note that the distortion comes from two different sources. First, the presence of *A*_*H*_ types in the closed-data regime allows the *A*_*L*_ types who choose that regime to falsify with some probability, which was impossible above and contributes to a larger overall rate. Also, however, a corollary to the argument above concerning the entry rate into the open regime is that the verification rate there must be lower than it would be in a single, open regime. Specifically, expression (11) implies that the threshold preparation cost $K_{L}^{*}$ drives a wedge between the expected utilities of the two regimes for *A*_*L*_. Since in the single-regimes, expected utility was equal to precisely *L* in both regimes, and the *k*-game in the closed regime implies that this remains the case in the current model, it follows that expected utility must be higher for *A*_*L*_ in the open regime. This then requires that the verification level be lower than its single-regime level.

Furthermore, it is immediate that a reduction in the preparation cost distribution—for instance in the sense of stochastic dominance—would reduce the levels of $\left(K_{L}^{*},K_{H}^{*}\right)$ that satisfy (9), (10), and (11), and therefore reduce this distortion, increasing science welfare. Indeed, the model in section “The Effect of Different Possible Levels of *k*” can be taken as a limiting case of that in section “Costs of Preparing Open Data,” when costs are reduced to zero. The result is therefore as follows:

*Result 5: Science welfare with preparation costs is reduced both by the entry of high-quality work into the closed data regime, and also by distortions of the verification rate in the open data regime.*

*Corollary 3: A leftward shift in the distribution of preparation costs will reduce these distortions and increase science welfare.*

## Discussion

The model in this paper investigated ways in which open data can leverage social esteem to discipline the reporting of scientific results. The key assumptions were (1) authors get a direct utility benefit from the public (equilibrium) perception of the quality of work they do; (2) readers get some utility benefit from discovering that the presented quality of a given result is inaccurate; (3) discovery of inflated inaccuracy, in which low-quality results are presented as high, imposes a utility cost on authors; (4) readers must incur a cost in order to check the accuracy of the presented results. These assumptions were selected to reflect potentially important elements of the publishing process, and set up a model in which open data—one of whose primary goals is to reduce the cost to readers of replicating or recreating published results—could have an influence on the tendency to misrepresent.

The model can be seen as an application of signaling games to the case of scientific publications. While this is not a specific subject that has received much theoretical treatment, signaling games generally represent of course a vast and rich field, from which much more is taken for this paper than is contributed. The structure of simple signaling games is very standard, and has been well-understood since Spence (1973); the application here used standard refinements such as sequential equilibrium (Kreps and Wilson, 1982a,b) and, to a limited extent the Intuitive Criterion (Cho and Kreps, 1987). The idea that the signal is designed to represent some otherwise unobservable quality that matters to the signal receiver also indicates links, for instance, to literature on advertising (see Bagwell, 2007). A modest theoretical innovation, designed to reflect the esteem-based nature of the benefit to the author of discovering important results, sees utility in the model as based directly on beliefs about the signal sender’s type, rather than—as is perhaps more common in economic interactions—based on the receiver’s reaction to those beliefs. But as mentioned, this is essentially a difference in interpretation and has little influence on the formal structure of the game.

Another departure from the standard signaling game that might be found in any advanced microeconomics course is the fact that in this model, there are effectively two sequential signals. Section “Selective Reporting and Verification Given Verification Costs” of the paper described a semi-separating *k-*game equilibrium in which authors of low-quality work partially imitated high quality, and showed that the lower the cost of verification, the less falsification there will be. A measure of *science welfare loss*, defined as the equilibrium level of unverified falsification of results, was found to be decreasing in the cost of verification. Section “The Effect of Different Possible Levels of *k*” then extended this to a case in which there were two possible levels of this cost or regimens—reflecting open and closed data—in which case the choice of one regime or the other could be seen as a second level of signaling. It found that while some low-quality work might use the high-cost signal, there was partial “unraveling” in that some low-quality work would also be presented with a low verification cost. This is basically a second level of semi-separation in regime choice. Interestingly, while behaviorally the model in section “The Effect of Different Possible Levels of *k*” did not pin down what the equilibrium distribution of low-quality work signals would be, the science welfare was the same regardless of whether the high-cost regime existed or not. In terms of the equilibrium level of distortion, open data completely crowded out closed.

In the model from section “The Effect of Different Possible Levels of *k*,” both the quality signal and the regime choice were essentially *cheap talk*, imposing no costs on the authors who chose them. In line with survey data and introspective evidence, section “Costs of Preparing Open Data” then extended the model to make using the open data regime costly relative to the closed. This resulted in some high-quality work being submitted in each regime, and increased the science welfare loss proportionally.

What does this model tell us about open data as a tool for strengthening the scientific publishing process? First, to the extent that readers get some benefit from correcting mistakes they find in the literature, facilitating this with open data should act as a disciplining tool for the presentation of results. Open data, in other words, should “work.” Furthermore, while the interpretation of player *B* in the model is as a representative reader who may spend effort to check results of published work, it is worth mentioning that any other effect that reduced the cost of close inspection of results should have similar effects. For instance, incentives for careful reviewing at the peer review stage, or institutional procedures on the part of employers or scientific journals could be implemented to reduce the opportunity cost of verification.^7^ Second, however, the model shows that this relies on the costs of preparing open data not being too high. In particular, the more high-quality work that is submitted in closed data, the greater the science welfare loss in equilibrium. Conversely, if the preparation costs are pushed down to zero, there is no need to impose open data on the scientific community; high-quality work will select into the low-verification-cost regime, and the residual work that goes into the high-cost regime will not affect the overall level of distortion in the literature, although interestingly, the few low-quality results that are published in open data will be more likely to be falsified when they are in a “stronger pool.”

The theoretical results from Sections “The Effect of Different Possible Levels of *k*” and “Costs of Preparing Open Data” both predict an overall correlation between the adoption of open data and research quality. This fits well with the existent empirical literature showing that papers published under open data have higher citation counts than those without (Pienta et al., 2010; Marwick and Birch, 2018). The results in these papers are correlational, and it is conceivable that the open data itself increased citation count through encouraging others to build on the published results—indeed that is the preferred interpretation in the literature. To this extent, the model is useful in supplying a justification for a separate causal interpretation of the data (see Soeharjono and Roche, 2021; for an early formal treatment see Verrecchia, 1990).

One of the more interesting implications these results may have concerns educational policies. Preparing data for open publication requires a specific set of skills, and explicitly training young academics in these skills seems bound to reduce their cost to doing so later. From the perspective of the model in section “Costs of Preparing Open Data,” this would result in the kind of “leftward shift” in the function *G*(*K*) that would reduce the equilibrium distortion rate. Similarly, part of the training in empirical work could be specifically in replicating existing studies using open data, or performing meta-analyses. Such measures would have the effect in the model of reducing *k* in any regime, which would increase verification rates and reduce falsification in all of them, again improving science welfare. Measures such as these might be better even than imposing open data on publication in the field. Even well-prepared data after all can only be verified by willing *B*-players. Also, the costs to verification and data preparation should be taken into account in a wider welfare criterion. Although equilibrium verification implies that agents are at least as well off incurring those costs as not, their final utility will obviously be improved if the costs are lower. From an even broader, “libertarian paternalist” perspective it may also be preferable to develop a system in which agents choose the “right” actions for themselves than one in which they are forced to do so. Such an argument has philosophical merit, and also utilitarian appeal, as those who are forced to engage in any action will be the most likely to try to find loopholes to avoid it.

## Data Availability Statement

The original contributions presented in the study are included in the article/Supplementary Material, further inquiries can be directed to the corresponding author.

## Author Contributions

ES: construction and resolution of the theoretical model.

## Conflict of Interest

The author declares that the research was conducted in the absence of any commercial or financial relationships that could be construed as a potential conflict of interest.

## Publisher’s Note

All claims expressed in this article are solely those of the authors and do not necessarily represent those of their affiliated organizations, or those of the publisher, the editors and the reviewers. Any product that may be evaluated in this article, or claim that may be made by its manufacturer, is not guaranteed or endorsed by the publisher.

## Footnotes

1 Costly verification puts this model somehow between cheap talk games in which the message is unverifiable, and signaling games as in Spence (1973).

2 To compare to canonical education signaling, it would be as though an employer prefers to give a high wage to low-productivity employees and *vice versa*.

3 The equilibrium utility is *E* [*q* | *h*] = *pH* + (1 − *p*) *L*, and exactly one type (*A*_*H*_) is in the situation where, if they sent the message *l*, and *B* believed that only they would send that signal, then it would be optimal for them to do so, as *B* would verify that signal and *A*_*H*_ would gain utility *H*. However, this deviation is not itself an equilibrium, as *B* would in this case also verify the signal *h*, leading *A*_*L*_ to deviate.

4 The condition $p<\frac{C}{C+\varepsilon }$ serves formally to guarantee that *pϵ* < (1 − *p*) *C*, so the latter threshold is lower than the former.

5 As pointed out by a reviewer, since nothing “happens” in the model between *A*’s choices, they are strategically simultaneous even if separated temporally in practice. If, for instance, —due perhaps to preregistration—authors had to commit to a regime before observing *q*, one can conjecture that the results might be more strongly affected.

6 Naturally, high-cost regimes will still have higher falsification and lower verification rates than low-cost ones do.

7 The author thanks a reviewer for highlighting this consideration.
