# Supplementary material for: Esteemed Colleagues: A Model of the Effect of Open Data on Selective Reporting of Scientific Results
Source: Front Psychol. 2021 Oct 21;12:761168. doi: 10.3389/fpsyg.2021.761168 (PMC8566335; doi:10.3389/fpsyg.2021.761168)
Supplement: Supplementary file 1 [file Data_Sheet_1.DOCX]

# Appendix A: equilibrium structure of the Prestige Game

The structure to establish is as follows:

- If $k>\left( 1-p \right)C$ then there are only pooling equilibria.
- If $k<\left( 1-p \right)C$ then there is a unique semi-separating equilibrium in which *A_L_* mixes and *A_H_* chooses *h* with probability 1 – this is called the *k-game*
- If $k<p\epsilon$ then there is also a pooling equilibrium in which all *A*-types choose *l* and *B* verifies with probability 1.

We proceed in “nested cases”, showing (A) no full separation; (B) the conditions and structure of semi-separation; and (C) the conditions for pooling.

## A: Show there is no full separation in pure strategies.

Proof:

If $s_{H}=\left( 1-s_{L} \right)=1$, then $v_{h}=0$, so *A_L_* will deviate rather than get *L* in expected value by playing *l*. On the other hand, if $s_{H}=\left( 1-s_{L} \right)=0$, then $v_{h}=1$, so *A_L_* will deviate again, rather than get *L – C*, her lowest possible payoff.

## B: Calculate semi-separating mixed strategies. Show that if p is low enough, then either $\boldsymbol{s}_{\boldsymbol{L}}\boldsymbol{<}\boldsymbol{s}_{\boldsymbol{H}}\boldsymbol{=1}$ and $\boldsymbol{0=}\boldsymbol{v}_{\boldsymbol{l}}\boldsymbol{<}\boldsymbol{v}_{\boldsymbol{h}}$, or $\boldsymbol{v}_{\boldsymbol{h}}\boldsymbol{=}\boldsymbol{v}_{\boldsymbol{l}}\boldsymbol{=0}$.

Proof:

The strategy will be to first show it is impossible for both *s_H_* and *s_L_* to be non-degenerate if $v_{h}+v_{l}>0$. Together with the result from part A, this shows that exactly one type mixes. We then we show that type to be *A_L_*.

First, then, suppose towards a contradiction that both A-types mix. Then

$v_{h}H-v_{l}H=\left( 1-v_{l} \right)E\left[ q|l \right]-\left( 1-v_{h} \right)E\left[ q|h \right],$ (A1)

$v_{h}\left( L-C \right)-v_{l}L=\left( 1-v_{l} \right)E\left[ q|l \right]-\left( 1-v_{h} \right)E\left[ q|h \right].$ (A2)

This implies

$v_{h}H-v_{l}H=v_{h}\left( L-C \right)-v_{l}L,$

$\left( v_{h}-v_{l} \right)\left( H-L \right)=-v_{h}C,$ (A3)

So clearly $v_{h}<v_{l}$, implying $v_{l}>0$ and $v_{h}<1$, or both are equal to zero. However, if $v_{h}=v_{l}=0$, then mixing by *A* requires that $E\left[ q | l \right]=E\left[ q | h \right]$, so $s_{L}=s_{H}=s$. This is therefore a fully-pooling strategy in which the message gives no information about the true state of *q*, and we leave it for the next subsection

The case of $0\leq v_{h}<v_{l}\leq1$ implies the following inequalities for *B*’s optimal behavior:

$\frac{\left( 1-p \right)s_{L}}{ps_{H}+\left( 1-p \right)s_{L}}C\leq k$

$\frac{p\left( 1-s_{H} \right)}{p\left( 1-s_{H} \right)+\left( 1-p \right)\left( 1-s_{L} \right)}\epsilon\geq k.$

At most one of the inequalities must be strict, since full separation is impossible. Putting these together, we get

$\frac{\left( 1-p \right)s_{L}}{ps_{H}+\left( 1-p \right)s_{L}}C\leq\frac{p\left( 1-s_{H} \right)}{p\left( 1-s_{H} \right)+\left( 1-p \right)\left( 1-s_{L} \right)}\epsilon$ (A4)

Expression (A4) puts some bounds or restrictions on $s_{L}$ and $s_{H}$, for the other parameter values. Note that if $s_{L}=s_{H}$, then (A4) implies that $\left( 1-p \right)C\leq p\epsilon$, or written differently, $p\geq\frac{C}{\epsilon+C}$. Therefore, if $p<\frac{C}{\epsilon+C}$, then case $0\leq v_{h}<v_{l}\leq1$ implies that $s_{H}>s_{L}$. However, this means that $E\left[ q|h \right]>E\left[ q | l \right]$. By A1, this further implies

$v_{h}H-v_{l}H<\left( 1-v_{l} \right)E\left[ q|h \right]-\left( 1-v_{h} \right)E\left[ q|h \right],$

$\left( v_{h}-v_{l} \right)H<\left( v_{h}-v_{l} \right)E\left[ q|h \right],$

$H<E\left[ q|h \right],$

which is impossible.

*If* $p<\frac{C}{\epsilon+C}$ *then either at most one A-type mixes, or B never verifies any signals.*

Noting that the notion that $\epsilon$ is “small” implies that this condition does not restrict *p* very much, we will maintain the assumption in what follows that it holds.

Further, if one *A*-type mixes, then it must be *A_L_*, with *A_H_* choosing the signal *h*. To see this, we again make an analysis by cases. There are four possibilities, denoting the strategies for (*A_L_*,*A_H_*) as (*h*,m), (*l*,m), (m,*h*) and (m,*l*) with m standing for non-degenerate mixed strategies.

Case (*h*,m) cannot stand

*A_L_* chooses *h* and *A_H_* mixes. In this case, only *A_H_* chooses *l*, so $E\left[ q | l \right]=H$, and $v_{l}=1$. If $v_{h}=1$, then *A_L_* gets caught and will deviate earning *L* instead of *L – C*. If $v_{h}<1$, then $E\left[ q | h \right]<H$, so *A_H_* will deviate. (Note this will “eventually” lead to $v_{h}=1$.)

Case (*l*,m) cannot stand

*A_L_* chooses *l* and *A_H_* mixes. In this case, only *A_H_* chooses *h*, so $E\left[ q | h \right]=H$, and $v_{h}=0$. If $v_{l}>0$, then *A_L_* earns *L* with some probability and will deviate to *h* (again, this may well unravel further). $v_{l}=0$, then $E\left[ q | l \right]<H$, so *A_H_* will deviate.

Case (m,*l*) cannot stand

*A_L_* mixes and *A_H_* chooses *l*. In this case, only *A_L_* chooses *h*, so $E\left[ q | h \right]=L$, and $v_{h}=1$. *A_L_* gets caught and will deviate to $s_{L}=0$, earning (at least) *L* instead of *L – C*.

Case (m,*h*) is an equilibrium with $v_{l}=0$ and $0<v_{h}<1$

*A_L_* mixes and *A_H_* chooses *h*. Only *A_L_* chooses *l*, so $v_{l}=0$ and $E\left[ q | l \right]=L<E\left[ q | h \right]$. *A_H_* strictly prefers *h* to *l* regardless of $v_{h}$. *A_L_* is indifferent so long as $v_{h}\left( L-C \right)+\left( 1-v_{h} \right)E\left[ q | h \right]=L$. This necessarily implies $0<v_{h}<1$. The equilibrium is determined by solving

$v_{h}\left( L-C \right)+\left( 1-v_{h} \right)\left[ \frac{p}{p+\left( 1-p \right)s_{L}}H+\frac{\left( 1-p \right)s_{L}}{p+\left( 1-p \right)s_{L}}L \right]=L$ (A5)

and

$\frac{\left( 1-p \right)s_{L}}{p+\left( 1-p \right)s_{L}}C=k.$ (A6)

This yields

$$v_{h}\left( L-C \right)+\left( 1-v_{h} \right)\left[ \left( 1-\frac{\left( 1-p \right)s_{L}}{p+\left( 1-p \right)s_{L}} \right)H+\frac{\left( 1-p \right)s_{L}}{p+\left( 1-p \right)s_{L}}L \right]=L$$

$$v_{h}\left( L-C \right)+\left( 1-v_{h} \right)\left[ \left( \frac{C-k}{C} \right)H+\frac{k}{C}L \right]=L$$

$$\left( 1-v_{h} \right)\left[ \left( \frac{C-k}{C} \right)H+\frac{k}{C}L \right]=L\left( 1-v_{h} \right)+v_{h}C$$

$$\left( 1-v_{h} \right)\left[ \left( \frac{C-k}{C} \right)H-L\left( \frac{C-k}{C} \right) \right]=v_{h}C$$

$$\left( 1-v_{h} \right)\left( \frac{C-k}{C} \right)\left( H-L \right)=v_{h}C$$

$$\frac{\left( \frac{C-k}{C} \right)\left( H-L \right)}{C+\left( \frac{C-k}{C} \right)\left( H-L \right)}=v_{h}$$

And reintroducing this into (A5),

$$\frac{\left( \frac{C-k}{C} \right)\left( H-L \right)}{C+\left( \frac{C-k}{C} \right)\left( H-L \right)}\left( L-C \right)+\left( \frac{C}{C+\left( \frac{C-k}{C} \right)\left( H-L \right)} \right)\left[ \frac{p}{p+\left( 1-p \right)s_{L}}H+\frac{\left( 1-p \right)s_{L}}{p+\left( 1-p \right)s_{L}}L \right]=L$$

$$\left[ \frac{p}{p+\left( 1-p \right)s_{L}}H+\frac{\left( 1-p \right)s_{L}}{p+\left( 1-p \right)s_{L}}L \right]=\left( \frac{C+\left( \frac{C-k}{C} \right)\left( H-L \right)}{C} \right)\left[ \frac{C}{C+\left( \frac{C-k}{C} \right)\left( H-L \right)}L+\frac{\left( \frac{C-k}{C} \right)\left( H-L \right)}{C+\left( \frac{C-k}{C} \right)\left( H-L \right)}C \right]$$

$$\left[ \frac{p}{p+\left( 1-p \right)s_{L}}H+\frac{\left( 1-p \right)s_{L}}{p+\left( 1-p \right)s_{L}}L \right]=\left[ L+\frac{\left( \frac{C-k}{C} \right)\left( H-L \right)}{C}C \right]$$

$$\left[ \frac{p}{p+\left( 1-p \right)s_{L}}H+\frac{\left( 1-p \right)s_{L}}{p+\left( 1-p \right)s_{L}}L \right]=\frac{k}{C}L+\left( \frac{C-k}{C} \right)H$$

$$pH+\left( 1-p \right)s_{L}L=\left[ \frac{k}{C}L+\left( \frac{C-k}{C} \right)H \right]\left( p+\left( 1-p \right)s_{L} \right)$$

$$\frac{p}{\left( 1-p \right)}\frac{\left( H-\left[ \frac{k}{C}L+\left( \frac{C-k}{C} \right)H \right] \right)}{\left( \left[ \frac{k}{C}L+\left( \frac{C-k}{C} \right)H \right]-L \right)}=s_{L}$$

$$\frac{p}{\left( 1-p \right)}\frac{\left( -L+H \right)\frac{k}{C}}{\left( H-L \right)\left( \frac{C-k}{C} \right)}=s_{L}$$

$$\frac{p}{\left( 1-p \right)}\frac{k}{C-k}=s_{L}.$$

So the unique semi-separating equilibrium strategies are

- $s_{H}=1$
- $s_{L}=\frac{p}{\left( 1-p \right)}\frac{k}{C-k}$
- $v_{h}=\frac{\left( \frac{C-k}{C} \right)\left( H-L \right)}{C+\left( \frac{C-k}{C} \right)\left( H-L \right)}$
- $v_{l}=0$

Note that this works only if

$$\frac{p}{\left( 1-p \right)}\frac{k}{C-k}\leq1$$

$$k\leq\left( 1-p \right)C$$

*If exactly one A player type mixes, then it is A_L_, and A_H_ chooses signal h with probability 1. This is known as the* k-game.

## C: Pooling equilibria

There are two potential pure-strategy pooling equilibria in this game, in addition to the mixed-strategy pooling equilibria identified above. In any pooling strategy, the expected value of *q* given the equilibrium strategy *s* is $E\left[ q | s \right]=pH+\left( 1-p \right)L:=\bar{p}$. In a Perfect Bayesian Equilibrium (PBE) context, pure-strategy pooling equilibria put no restrictions on beliefs about signals not sent in equilibrium. The existence of pooling equilibria depends on (1) how *B* responds to $E\left[ q | s \right]=\bar{p}$, and (2) on what beliefs are determined for the off-path signals.

The optimal behavior on the equilibrium path is determined by the relative magnitudes of *k*, (1 – *p*)*C* and *p*$\epsilon$, and in particular on the place of *k* relative to the other two. Note that the assumption that $p<\frac{C}{\epsilon+C}$ implies $\left( 1-p \right)C>p\epsilon$, which means there are generally three cases: (i) $p\epsilon<\left( 1-p \right)C<k$; (ii) $p\epsilon<k<\left( 1-p \right)C$; or (iii) $k<p\epsilon<\left( 1-p \right)C$. In (i), the cost of verification is high enough that *B* will not verify pooled strategies on either signal; (ii) implies that *B* will verify pooled messages of *h* but not of *l*, while under (iii), pooling on either signal will generate verification by *B*. PBEs do not restrict beliefs off the equilibrium path, which is generally held to be insufficiently restrictive. To put more structure on the off-path beliefs, we will consider sequential equilibria (SE) in the sense of Kreps and Wilson (1982). These suppose a sequence of perturbations around equilibrium strategies that converges to zero. The sequential equilibrium will be the equilibrium of the limit of this sequence. Equivalently, the analysis here will require beliefs such that in a sequence $S=\left\{ \tilde{s}_{H}\left( n \right), \tilde{s}_{L}\left( n \right) \right\}_{n=1}^{\infty}$ of “trembles” from the equilibrium strategies $s_{H}$ and $s_{L}$ such that $\left\{ \tilde{s}_{H}\left( n \right), \tilde{s}_{L}\left( n \right) \right\}\neq\left\{ s_{H},s_{L} \right\}$ for all *n*, but $\lim_{n\to\infty} S_{n}=\left\{ s_{H},s_{L} \right\}$, beliefs for each element of the sequence are determined by Bayes’ rule, and behavior is optimal given beliefs.

Consider case (i). When both $p\epsilon$ and $\left( 1-p \right)C$ are less than *k*, no pooling strategies are investigated by *B*, and either can be sustained as an equilibrium with, for instance, off-path beliefs of equal tremble probabilities for both *A*-player types. In fact, $\left( 1-p \right)C<k$ implies that condition 2 of the k-game cannot be satisfied for $s_{L}\leq1$. In this case even the k-game cannot be sustained in equilibrium: verification is too costly and all that remain are pooling equilibria.

*Under condition (i), only pooling equilibria are possible, and A may pool on either signal.*

If $v_{h}=v_{l}=0$, then mixing by *A* requires that $E\left[ q | l \right]=E\left[ q | h \right]$, so $s_{L}=s_{H}=s$. From *B*’s perspective, $v_{h}=v_{l}=0$ then requires both

$\frac{\left( 1-p \right)s}{ps+\left( 1-p \right)s}C=\left( 1-p \right)C<k$ (A5)

and

$\frac{p\left( 1-s \right)}{p\left( 1-s \right)+\left( 1-p \right)\left( 1-s \right)}\epsilon=p\epsilon<k$ (A6)

*If* $k>\max\left[ \left( 1-p \right)C,p\epsilon\right]$*, then there is a continuum of “babbling” pooling equilibria in which both types of A-player behave identically, and B never verifies.*
