# Supplementary material for: Esteemed Colleagues: A Model of the Effect of Open Data on Selective Reporting of Scientific Results
Source: Front Psychol. 2021 Oct 21;12:761168. doi: 10.3389/fpsyg.2021.761168 (PMC8566335; doi:10.3389/fpsyg.2021.761168)
Supplement: Supplementary file 2 [file Data_Sheet_2.docx]

Appendix B: Proof of Result 2

Result 2: Equilibrium under regime choice

*Consider a 2-regime prestige game where the k-game conditions hold in each regime, k_o_ < k_c_, and define λ and φ as in the paper. In any equilibrium*

1. ${\lambda_{k}}_{o}\left( H \right)=1$*;* $\frac{p}{\left( 1-p \right)}\frac{k_{o}}{C-k_{o}}< \lambda_{k_{o}}\left( L \right)\leq1$
2. *Behavior follows the k-game in the k_o_ regime; A_L_ always plays l in k_c_*
3. *Off-path beliefs mimic the k-game in the k_c_ regime*

The equilibrium in Result 2 can be established constructing it as an equilibrium candidate, and showing that the other conceivable candidates are not tenable. In general, there could be (a) two separate *k*-games, one in each regime; (b) complete *regime-separation* in which each type chooses a different regime; (c) *regime-semiseparation* in which one type always plays one regime and the other mixes across them (as in Result 2); (d) *regime-pooling* in which all *A*-types choose the same regime. Cases (a) and (b) are impossible, and case (c) takes the form outlined in Result 2. Case (d) is in fact a limiting special case of (c) in which the mixing rate of the second type puts all weight on one regime/

To begin, consider the following lemma:

Lemma 1:

*There is no interior (i.e. non-degenerate) mixed equilibrium where some fraction of H-type A players choose k_o_, and a (possibly different) fraction of L-types do.*

That is, there cannot be two simultaneous *k*-games as described in Result 1. To see this, note towards a contradiction that it would require not just mixing across signals for *A_L_*, but also mixing over regimes for both types, which implies that *A* players of each type must be indifferent across regimes. This is not problematic for *A_L_* – indeed, the mixed strategy that *A_L_* follows implies that in any *k*-game, the expected utility is precisely *L*. But it cannot hold for both types. If it did, we would have

$v_{o}H+\left( 1-v_{o} \right)E\left[ q|h,k_{o} \right]=v_{c}H+\left( 1-v_{c} \right)E\left[ q|h,k_{c} \right]$ (B1)

$v_{o}\left( L-C \right)+\left( 1-v_{o} \right)E\left[ q|h,k_{o} \right]=v_{c}\left( L-C \right)+\left( 1-v_{c} \right)E\left[ q|h,k_{c} \right].$ (B2)

However, since *k_o_* < *k_c_* implies that $E\left[ q|h,k_{o} \right]>E\left[ q|h,k_{c} \right]$, (B1) requires *v_c_* > *v_o_*, while (B2) requires the reverse inequality to hold. This contradiction establishes the result.

Furthermore, (B2) does hold with equality in equilibrium, as both sides are equal to *L*, being the *k-*game components of their respective regimes. It follows that (B1) must be greater on the left-hand side. Intuitively, *A_H_* gets two benefits from *k_o_*: a higher verification rate (and hence probability of receiving a payoff of *H*); and also a higher expected return when verification does not occur. As a result, from this fully mixed equilibrium, the *A_H_* types will all deviate to *k_o_*. This establishes the second lemma, which guarantees that regime-semiseparation takes the form in Result 2:

Lemma 2:

*A_L_ is indifferent between regimes under the k-game; A_H_ strictly prefers the one with a lower cost*

Lemmas 1 and 2 establish the first clause of part A of result 2. *A_H_* chooses *k_o_* with probability 1 and *A_L_* mixes across regimes. Hence, the *k_c_* regime contains only results of quality *L*, and any attempt to falsify will be identified, establishing as optimal *A_L_*’s signal choice in that regime: *s_c_* = 0. As we saw in Remark 1, the fewer *A_L_*-types who enter the *k_o_* regime, the higher the falsification rate among those that return to maintain *B*’s equilibrium mixing in verification. But so long as

$$\lambda_{k_{o}}\left( L \right)>\frac{p}{\left( 1-p \right)}\frac{k_{o}}{C-k_{o}},$$

the equilibrium falsification rate is less than unity, and the *k*-game stands in the low-cost regime. Therefore the condition on $\lambda_{k_{c}}\left( L \right)$ in part A ensures that *A_H_*’s behavior in the *k_o_* regime remains optimal.

Finally, part C of Result 2 is complicated by the fact that signals of *h* in the *k_c_* regime are never observed in this equilibrium candidate, so off-path beliefs regarding how it would be interpreted are important to the analysis of potential deviations. The key point is whether *B* would verify a (nonequilibrium) signal of *h* in the *k_c_* regime or not. The point to establish can be stated

Lemma 3:

*Off-path beliefs that would lead B to verify a deviant signal of h with degenerate probability (equal to zero or one) upset the equilibrium in Result 2*

Suppose the beliefs lead to verification: *v_c_*(*h*) = 1. Then *A_H_* will deviate from the mixed equilibrium in *k_o_* to get the payoff *H* with probability 1 upon verification in *k_c_*. On the other hand, if *v_c_*(*h*) = 0, then then *A_L_* will deviate to give the signal *h* unless the expected value of that signal is precisely *L*, that is Pr[*L*|*h, k_c_*] = 1. But that is amply sufficient to ensure that *B* will in fact verify the results. *B*’s expected utility from verification is $\Pr\left[ L | h,k_{c} \right]C-k_{c}$, so *B* rationally chooses to verify if Pr[*L*|*h, k_c_*] > *k_c_*/*C* , which we assumed less than one at the outset. The result is therefore that if *B* verifies, then *A_H_* will deviate in regimes, but in any condition where *B* does not verify, *A_L_* will deviate in messages within the regime *k_c_*. This establishes the result.

However, if *B* verifies at precisely the level required to make *A_L_* indifferent between regimes – that is, at the level determined in the high-cost *k*-game – then *A_L_* can still follow the equilibrium, and *A_H_* strictly prefers *k_o_* to *k_c_*. Therefore, beliefs that recreate the high-cost *k*-game off the equilibrium path support the equilibrium above. The off-path beliefs held by *B* to make this work appear rather precise. Denoting off-path beliefs with bars, *B*’s probabilistic verification relies on the condition that

$$\frac{\bar{s}_{c}\left( 1-p \right)\lambda_{k_{c}}\left( L \right)}{\bar{s}_{c}\left( 1-p \right){\lambda_{k}}_{c}\left( L \right)+p\bar{\lambda}_{k_{c}}\left( H \right)}C=k_{c}.$$

Since in equilibrium, both *s_c_* and $\lambda_{k_{c}}\left( H \right)$ are equal to zero, this belief requires *B* to conjecture two simultaneous kinds of deviation to explain any off-path observations of *h* in *k_c_*. Indeed, the ratio of *B*’s beliefs about $\bar{s}_{c}$ and $\bar{\lambda}_{k_{c}}\left( H \right)$ will be determined by the equilibrium value of $\lambda_{k_{c}}\left( L \right)$.

$$\frac{\bar{\lambda}_{k_{c}}\left( H \right)}{\bar{s}_{c}}=\lambda_{k_{c}}\left( L \right)\frac{\left( 1-p \right)\left( C-k_{c} \right)}{pk_{c}}$$

These off-path beliefs admit an interesting interpretation in terms of “non-stigmatization”. In essence, while the equilibrium never has high-quality results in the *k_c_* regime, it relies on the belief that this “could happen” off the equilibrium path, and so signals of type *h* in *k_c_* are not systematically verified. On the other hand, *B* would also ascribe such a “tremble” to *A_L_* types with some probability, so some verification occurs. In other words, the unobserved, counterfactual declarations of *h* in the high-cost regime are not given particularly bad (or good) interpretations in the model.

Taken together, these results give

Lemma 4:

*There exists a continuum of “partial pooling” equilibria in which, (1) A_H_ always chooses k_o_ and h; (2) A_L_ chooses k_o_ with probability* $\frac{p}{\left( 1-p \right)}\frac{k_{o}}{C-k_{o}}< \lambda_{k_{o}}\left( L \right)\leq1$*, and then s_o_ = s_o_^*^ and s_c_ = 0 in the respective regimes; (3) off the equilibrium path, B expects behavior consistent with the k-game in k_c_*

The lower bound on the participation of *A_L_* in the low-cost regime was to ensure the existence of falsification rates consistent with *B*’s mixed strategy. This is crucial to the equilibrium results. If there are not enough papers of quality *L* in the *k_o_* regime, *B* does not verify the results, and then *A_L_* strictly prefers pooling with *A_H_* – incidentally destabilizing the beliefs on which the effect is based. As a special case, consider the “separating-regime” equilibrium candidate in which each *A*-type chooses a different regime. *B* will not verify the regime chosen by *A_H_*, which does not matter to *A* since in this equilibrium all messages in that regime are taken as quality *H*. But it will lead *A_L_* to deviate, imitating *A_H_* and undoing the equilibrium. The following is therefore essentially a corollary of Lemma 2, and rules out the case of regime-separation.

Corollary 1: The model has no equilibria in which A-types separate in regimes

Finally, note that the bounds on $\lambda_{k_{c}}\left( L \right)$ admit the possibility that all *A*-types choose *k_o_*. With the same off-path beliefs of *B* described above, therefore, “pure pooling” on *k_o_* is also an equilibrium of this game. This is supported by the fact that *A_L_* is indifferent between the regimes, earning *L* on average no matter what, while *A_H_* strictly prefers *k_o_*. For that reason, the opposite pool on *k_c_* is not an equilibrium.

Corollary 2: The case where $\lambda_{k_{c}}\left( L \right)=0$ represents a “pure pooling” special case of the equilibrium

This establishes the Result 2.
